# Higher Frequency of Community-Based Group Exercises in Temporary Communities Compared With Original Communities After the Great East Japan Earthquake: A Cross-Sectional Study

**DOI:** 10.7759/cureus.109576

**Published:** 2026-05-24

**Authors:** Atsushi Matsunaga, Chie Teramoto, Satoko Nagata

**Affiliations:** 1 Department of Public Health Nursing, Division of Health Sciences, Graduate School of Medicine, Tohoku University, Sendai, JPN; 2 Department of Perioperative and Critical Care Management, Graduate School of Biomedical and Health Sciences, Hiroshima University, Hiroshima, JPN; 3 Faculty of Nursing and Medical Care, Keio University, Fujisawa, JPN

**Keywords:** community health nursing, community organization, community participation, evacuation, exercise, great east japan earthquake, japanese geriatrics, relocation, social capital, temporary housing

## Abstract

Introduction: This study aimed to conduct an exploratory investigation to compare the monthly implementation frequency of community-based group exercises between mildly affected communities (original communities) and severely affected communities (temporary communities) in a municipality affected by the Great East Japan Earthquake in 2011.

Methods: A cross-sectional survey was conducted from February to March 2014, in Otsuchi town, one of the municipalities most severely damaged by the earthquake. Participants were 85 leaders who were in charge of either an original or a temporary community. The questions pertained to the participants themselves and the frequency of community-based group exercises conducted in their communities in January 2014.

Results: Of the 85 community leaders invited, 32 (37.6%) provided usable responses. Community-based group exercises in temporary communities were more frequent than in original communities. The community residents themselves hosted group exercises more frequently in temporary communities than did those in original communities.

Conclusion: These exploratory findings suggest that residents in temporary communities may have demonstrated greater initiative in organizing community-based group exercises than those in original communities. This may be attributed to the severity of the disaster's impact and the support for community organizing provided during the recovery phase. Community and public health professionals need to actively promote community organizational activities after a disaster to decrease the likelihood of mental and physical health problems.

## Introduction

On March 11, 2011, the Great East Japan Earthquake and an accompanying tsunami struck northeastern Japan. As a result, by May 9, 2019, 19,667 people had died, 2,566 people were missing, and 1,159,613 houses were damaged. This included 121,783 houses that were completely destroyed [1]. The Japanese government established 53,537 prefabricated temporary housing structures in and around the affected areas, mainly on land with no buildings, such as fields, athletic fields, school grounds, and so on. From July to August 2011, many affected people who had lost their homes relocated from their original communities to temporary communities where the government had established prefabricated temporary housing, while permanent housing was being repaired and completed [2].

After such a major disaster, a wide range of physical and mental health problems arise, including post-traumatic stress disorder, depression, congestive heart failure, diabetes, hypertension, and respiratory conditions [3-5]. Disasters have also been associated with declines in activities of daily living (ADL) and instrumental activities of daily living (IADL), in particular among older adults [6], as reported following the Bam earthquake in Iran [7] and the Great East Japan Earthquake [8]. Such declines in ADL and IADL are often mediated by deterioration in physical and mental health and by social disruption [6,8].

To address these health problems, the implementation of group exercises (physical activities performed with at least one other person) has been observed to reduce the risk of physical and mental illness by improving adherence to physical activity, psychological factors, and social relationships [9]. Therefore, for community and public health professionals working in disaster-affected areas, actively promoting participation in group exercise is essential as an important public health strategy to safeguard and improve the physical and mental well-being of affected populations.

Previous literatures reported group exercises conducted within communities and targeting community residents (hereafter, community-based group exercises) in disaster-affected communities. For example, Tomata et al. [10] reported that participants of these group exercises showed improved self-rated health and were more likely to go out compared with non-participants. However, little is known about how frequently these group exercises have been conducted in disaster-affected communities. The living environments and social structures differ significantly between temporary communities, where residents experienced severe loss and relocation, and original communities, where residents remained in their homes with relatively less damage. We hypothesized that these distinct situational contexts would result in different implementation frequencies and, crucially, different types of organizing entities (e.g., resident-led vs. agency-led) for community-based group exercises. Identifying these differences is essential for understanding the current status of community activities and for determining the specific support required in various post-disaster settings. Therefore, the study objective was to conduct an exploratory investigation to examine the monthly implementation frequency of community-based group exercises, defined as physical activities performed with at least one other person, conducted within local communities, and targeting community residents, and to compare these implementation frequencies between severely affected temporary communities and less affected original communities through a survey in a municipality affected by the Great East Japan Earthquake.

## Materials and methods

Research design and setting

Two years and 11 months after the earthquake, from February to March 2014, we conducted a cross-sectional survey in Otsuchi town, in the Iwate prefecture of Japan. The municipality was one of the areas most severely damaged by the earthquake and the tsunami. Of its total population, 1284 people (8%) died, 4.31 km^2^ (52% of residential area) was flooded, and 4,090 houses (62.9%) were damaged, including 3,374 houses (51.9%) that were completely destroyed [11,12]. As a result of this devastation, the national and local government established 48 temporary communities and 2,106 temporary housing structures in the municipality [11]. Then, from the end of April to the beginning of August 2011, 4,769 affected persons relocated to these temporary dwellings from evacuation centers [11]. As of January 31, 2014, when the survey was conducted, 4,144 affected people remained in these temporary locations [13]. 

All communities in Otsuchi town were affected by the earthquake, the tsunami, and an accompanying, large-scale fire. However, the degree of the damage varied among communities. For example, in the communities that were not flooded by the tsunami, the residents were able to continue to live in their houses after the earthquake. We refer to these communities as original communities. In the communities that suffered serious damage from the earthquake, the tsunami, and the fire, residents lost their houses and relocated to prefabricated temporary dwellings in newly established communities. We refer to these communities as temporary communities.

Given these differences in damage and living conditions across communities, two types of personnel were deployed: life support advisors (LSAs) and community support staffs (CSSs). LSAs, who were generally not specialists in social work, medicine, or healthcare, were assigned to the local Social Welfare Council to support residents across the entire town, including both original and temporary communities. Their primary responsibilities included home visits, health consultations, and health promotion activities throughout the municipality.

In contrast, CSSs were recruited from among affected residents through a project commissioned by Otsuchi town to support the management of temporary housing complexes. Their responsibilities, limited to these temporary settings, included facility maintenance, community monitoring, coordinating volunteer activities, and facilitating communication between residents and public agencies.

Participants and study procedure

Participants were 85 leaders of both the original and the temporary communities. These included leaders of the residents' associations and community welfare volunteers, who best understood the contexts of their population. We entrusted the Otsuchi town office to mail self-reported questionnaires to these leaders in February 2014. Of the 85 leaders, 32 (37.6%) answered the questionnaires, sealed them in envelopes, and mailed them back to us or put them in a collection box installed at the Otsuchi town office in March 2014.

Instruments

We created a questionnaire (see Appendices) by means of iterative consultations with public health nurses belonging to the Otsuchi town office to ensure the content validity and contextual appropriateness of the items for the post-disaster setting. These public health nurses, who were deeply familiar with the local recovery context and the residents' living conditions, reviewed the questionnaire items for clarity and relevance. In the questionnaire, for each of the original and temporary communities, we asked the participants whether they were in charge of their communities and, if so, for how long. We then focused on questions regarding the community-based group exercises conducted during January 2014, two years and 10 months after the earthquake. If the participants replied that they were not in charge, they were not requested to answer further questions about their respective community.

Questions on community-based group exercises assessed overall frequency and the frequency by organizing group: neighborhood associations; resident groups other than neighborhood associations; CSSs, LSAs, or related personnel; the municipal government or organizations commissioned by it; external organizations (e.g., non-governmental organizations (NGOs) and universities); and other organizations, groups, or individuals. Responses regarding the frequency of these exercises were scored on a six-point Likert scale: almost every day, three to four times a week, once to twice a week, two to three times a month, once a month, or none.

Community-based group activities organized by different types of groups were often implemented in a complementary manner. For example, in communities where residents actively organized many group activities, neighborhood organizations tended to reduce the frequency of similar activities, perceiving less need to duplicate such efforts. To account for these characteristics, we aggregated the data by the characteristics of the organizing group. "Neighborhood associations" and "resident groups other than neighborhood associations" were combined as "community residents". "CSSs, LSAs, or related personnel" and "municipal government or organizations commissioned by it" were grouped as "public agencies". "External organizations" and "other organizations, groups, or individuals" were categorized as "private agencies from outside the community". To preserve data rigor, the highest reported frequency among the original variables was used as the aggregated value. This approach ensured that the results reflected the frequency of exercise opportunities that were being implemented at the very least within each category. Since events co-hosted by multiple organizers were recorded under each respective organizing group, summing the frequencies would have resulted in the same event being counted multiple times. By adopting the highest frequency instead of summing them, we prevented overestimation due to potential double-counting of collaborative events, thereby providing a baseline measure of sustained community activity.

In addition, changes in the frequency of community group activities between January 2013 and January 2014 were assessed using a three-point Likert scale (increased, no change, or decreased), without distinguishing between types of organizing groups.

Statistical methods

After confirming the descriptive statistics and distribution of the data, we conducted statistical tests to compare the original and temporary communities. Fisher's exact test was used to compare categorical variables, such as the geographical distribution of districts. For ordinal variables, including the frequency of community-based group exercises, Mann-Whitney U tests were performed. U-statistics were calculated based on group sizes and Z-values obtained from JMP® 19 (JMP Statistical Discovery LLC, Cary, North Carolina, United States). Regarding missing values, no data imputation was performed, and they were excluded from the analysis on an item-by-item basis; all returned questionnaires were included as no completely invalid responses were identified. All statistical analyses were conducted using JMP® 19. The statistical significance was set at p<0.05.

Regarding the independence of observations, nine community leaders provided data for both an original and a temporary community. These responses were treated as independent observations because the analysis focused on community-level characteristics and activity frequencies rather than on the individual leaders themselves. The exercises in each community type were distinct occurrences involving different resident groups and settings.

Ethics

All study procedures were examined and approved by the Research Ethics Committee of the Faculty of Medicine of the University of Tokyo (approval number: 10306). Through a study leaflet enclosed with the survey, we explained to the survey participants that the participation was voluntary and that refusing to participate would not result in negative consequences. Consent to participate in the survey was assumed when respondents read, filled out, and returned the questionnaire.

## Results

The characteristics of the participants are detailed in Table 1. Of the 85 community leaders invited, 32 (37.6%) provided usable responses to the questionnaire. Of these, 17 (53.1%) led temporary communities only, nine (28.1%) managed both original and temporary communities, and six (18.8%) were responsible for original communities only. The average duration of leadership was 26.6±4.4 months in original communities and 64.0±52.0 months in temporary communities. Regarding the geographical distribution of the communities, no significant differences were observed between original and temporary communities across the four districts (p=0.968; Table 2).

**Table 1:** Characteristics of the participants (n=32)

Characteristic	n (%) or mean±SD
The community in charge as a leader	
Original communities only	6 (18.8)
Temporary communities only	17 (53.1)
Both	9 (28.1)
Average charge period of communities (months)	
Original community	26.6±4.4
Temporary community	64.0±52.0

**Table 2:** Districts of participants' assigned communities Statistical analysis: The p-value was calculated using Fisher's exact test to compare the distribution of districts between original and temporary communities. Significance: Statistical significance was set at p<0.05. Rounding: Percentages may not total 100% due to rounding. Anonymization: District names (A-D) have been masked to protect the anonymity of the participants.

District	Total (n=41) n (%)	Original (n=15) n (%)	Temporary (n=26) n (%)	p
A	10 (24.4)	3 (20)	7 (26.9)	0.968
B	13 (31.7)	5 (33.3)	8 (30.8)	
C	12 (29.3)	5 (33.3)	7 (26.9)	
D	6 (14.6)	2 (13.3)	4 (15.4)	

Frequency of community-based activities

Three clear patterns were observed in the frequency of community-based group exercises across the three analytical levels (Table 3).

**Table 3:** Frequency of community-based activities in original communities and temporary communities CSSs: community supporting staffs; LSAs: life support advisors; NGOs: non-governmental organizations Statistical analysis: The p-values were calculated using the Mann-Whitney U tests to compare the frequency distributions between original and temporary communities. Significance: Statistical significance was set at p<0.05. Data aggregation: To determine the frequency for aggregated categories, the higher frequency between the respective sub-groups was adopted: Community residents: The higher frequency between "neighborhood associations" and "resident groups other than neighborhood associations". Public agencies: The higher frequency between "CSSs, LSAs, or related personnel" and "municipal government or organizations commissioned by it". Private agencies from outside of the communities: The higher frequency between "external organizations" and "other organizations, groups, or individuals".

Organizing group and frequency	Total (n=41) n (%)	Original (n=15) n (%)	Temporary (n=26) n (%)	U	p
All community-based group exercises					
Almost every day	11 (26.8)	1 (6.7)	10 (38.5)	82.4	0.002
Three to four times a week	6 (14.6)	1 (6.7)	5 (19.2)		
Once to twice a week	2 (4.9)	1 (6.7)	1 (3.8)		
Twice to three times a month	2 (4.9)	0 (0)	2 (7.7)		
Once a month	8 (19.5)	4 (26.7)	4 (15.4)		
None	12 (29.3)	8 (53.3)	4 (15.4)		
By the organizing group					
Neighborhood associations					
Almost every day	4 (9.8)	0 (0)	4 (15.4)	131.4	0.085
Three to four times a week	0 (0)	0 (0)	0 (0)		
Once to twice a week	0 (0)	0 (0)	0 (0)		
Twice to three times a month	3 (7.3)	0 (0)	3 (11.5)		
Once a month	7 (17.1)	3 (20)	4 (15.4)		
None	27 (65.9)	12 (80)	15 (57.7)		
Resident groups other than neighborhood associations					
Almost every day	4 (9.8)	0 (0)	4 (15.4)	146.3	0.187
Three to four times a week	2 (4.9)	0 (0)	2 (7.7)		
Once to twice a week	4 (9.8)	2 (13.3)	2 (7.7)		
Twice to three times a month	1 (2.4)	0 (0)	1 (3.9)		
Once a month	4 (9.8)	2 (13.3)	2 (7.7)		
None	26 (63.4)	11 (73.3)	15 (57.7)		
CSSs, LSAs, or related personnel					
Almost everyday	5 (12.2)	1 (6.7)	4 (15.4)	130.9	0.083
Three to four times a week	1 (2.4)	0 (0)	1 (3.9)		
Once to twice a week	2 (4.9)	1 (6.7)	1 (3.9)		
Twice to three times a month	6 (14.6)	0 (0)	6 (23.1)		
Once a month	4 (9.8)	2 (13.3)	2 (7.7)		
None	23 (56.1)	11 (73.3)	12 (46.2)		
Municipal government or organizations commissioned by it					
Almost every day	0 (0)	0 (0)	0 (0)	175.5	0.597
Three to four times a week	0 (0)	0 (0)	0 (0)		
Once to twice a week	1 (2.4)	0 (0)	1 (3.9)		
Twice to three times a month	5 (12.2)	2 (13.3)	3 (11.5)		
Once a month	10 (24.4)	3 (20)	7 (26.9)		
None	25 (61)	10 (66.7)	15 (57.7)		
External organizations (e.g., NGOs and universities)					
Almost every day	0 (0)	0 (0)	0 (0)	165.3	0.421
Three to four times a week	0 (0)	0 (0)	0 (0)		
Once to twice a week	1 (2.4)	0 (0)	1 (3.9)		
Twice to three times a month	1 (2.4)	0 (0)	1 (3.9)		
Once a month	6 (14.6)	2 (13.3)	4 (15.4)		
None	33 (80.5)	13 (86.7)	20 (76.9)		
Other organizations, groups, or individuals					
Almost every day	0 (0)	0 (0)	0 (0)	122.6	0.070
Three to four times a week	0 (0)	0 (0)	0 (0)		
Once to twice a week	1 (2.5)	1 (6.7)	0 (0)		
Twice to three times a month	0 (0)	0 (0)	0 (0)		
Once a month	1 (2.5)	1 (6.7)	0 (0)		
None	38 (95)	13 (86.7)	25 (100)		
By the characteristics of the organizing group					
Community residents					
Almost every day	7 (17.1)	0 (0)	7 (26.9)	120.5	0.044
Three to four times a week	2 (4.9)	0 (0)	2 (7.7)		
Once to twice a week	3 (7.3)	2 (13.3)	1 (3.8)		
Twice to three times a month	2 (4.9)	0 (0)	2 (7.7)		
Once a month	6 (14.6)	3 (20)	3 (11.5)		
None	21 (51.2)	10 (66.7)	11 (42.3)		
Public agencies					
Almost every day	5 (12.2)	1 (6.7)	4 (15.4)	143.0	0.159
Three to four times a week	1 (2.4)	0 (0)	1 (3.8)		
Once to twice a week	2 (4.9)	1 (6.7)	1 (3.8)		
Twice to three times a month	9 (22)	2 (13.3)	7 (26.9)		
Once a month	5 (12.2)	2 (13.3)	3 (11.5)		
None	19 (46.3)	9 (60)	10 (38.5)		
Private agencies from outside of the communities					
Almost every day	0 (0)	0 (0)	0 (0)	187.0	0.829
Three to four times a week	0 (0)	0 (0)	0 (0)		
Once to twice a week	2 (4.9)	1 (6.7)	1 (3.9)		
Twice to three times a month	1 (2.4)	0 (0)	1 (3.9)		
Once a month	7 (17.1)	3 (20)	4 (15.4)		
None	31 (75.6)	11 (73.3)	20 (76.9)		

First, the overall frequency of community-based group exercises was significantly higher in temporary communities than in original communities (p=0.002). Specifically, 38.5% (n=10) of participants from temporary communities reported that such exercises were conducted almost daily, whereas 53.3% (n=8) of participants from original communities reported that no such activities were conducted.

Second, when examining exercise frequency by individual organizing group, no statistically significant differences were observed between original and temporary communities for any specific group. These included exercises organized by neighborhood associations (p=0.085), resident groups other than neighborhood associations (p=0.187), CSSs or LSAs (p=0.083), municipal governments (p=0.597), external organizations such as NGOs and universities (p=0.421), and other organizations, groups, or individuals (p=0.070).

Third, when organizing groups were aggregated by their characteristics, activities led by "community residents" were conducted significantly more frequently in temporary communities than in original communities (p=0.044). In temporary communities, 26.9% (n=7) of these resident-led activities were conducted almost daily, whereas no such cases were reported in original communities (0%). In contrast, no significant differences were observed in activities organized by public agencies (p=0.159) or external private agencies (p=0.829).

Change in frequency of community-based group exercise

Table 4 shows the perceived changes in group exercise frequency between January 2013 and January 2014. Most participants in both community types reported no change in group exercise frequency (p=0.907). Specifically, seven participants (53.9%) from original communities and 12 participants (46.2%) from temporary communities reported that group exercise frequency remained stable. These findings suggest that the higher frequency of group exercise observed in temporary communities was not a transient increase or an anomaly unique to the timing of the survey.

**Table 4:** Change in the frequency of community-based group exercise Statistical analysis: The p-value was calculated using Fisher's exact test to compare the perceived change in exercise frequency between original and temporary communities. Significance: Statistical significance was set at p<0.05. Rounding: Percentages may not total 100% due to rounding.

Change in frequency ​​​​​​	Total (n=39) n (%)	Original (n=13) n (%)	Temporary (n=26) n (%)	p
Increase	9 (23.1)	3 (23.1)	6 (23.1)	0.907
No change	19 (48.7)	7 (53.9)	12 (46.2)	
Decrease	11 (28.2)	3 (23.1)	8 (30.8)	

## Discussion

We examined the frequency of community-based group exercise in original and temporary communities within a municipality severely affected by the Great East Japan Earthquake. No significant differences were observed between community types in the frequency of group exercise held by public agencies or external private organizations. However, group exercise held by residents was conducted significantly more frequently in temporary housing communities. When all three types were combined, the overall frequency of group exercise was also significantly higher in temporary housing communities. These results reveal that temporary community residents implemented community-based group exercises more frequently than residents in original communities. Furthermore, the fact that 48.7% of participants answered that the frequency of community-based group exercises had not changed in January 2014, compared with January 2013, suggests that the findings in early 2014 were not merely a temporary phenomenon but reflected an established level of activity during that period of the recovery phase. In this section, we will explore possible explanations for the higher reported frequency of community-based group exercises in temporary communities compared with original communities.

There are two possible explanations for the higher reported frequency of community-based group exercises in temporary communities compared with those in original communities. First, the extent of earthquake damage may have stimulated community engagement, leading to more frequent activities. Yamamura [14] demonstrated that the likelihood of participating in community activities increased following the Great Hanshin-Awaji Earthquake, particularly among individuals living in severely affected areas. This research further suggested that although such disasters damage physical assets and existing social ties, they also promote collective action and the formation of new social networks in preparation for future disasters [14]. In line with this perspective, the extensive damage caused by the Great East Japan Earthquake may have stimulated efforts among residents in temporary communities to build new social capital, such as resident-led group exercise within those communities.

Second, the support provided to each community after the disaster may facilitate the implementation of resident-led community-based group exercises. Disasters such as the Great East Japan Earthquake disrupt social relationships as a result of the death of family members, friends, and neighbors. Further disruptions can be caused by the responses to the disaster, such as evacuation, breakdowns in transportation, failure of communication systems, and relocation [15]. People living in temporary communities tend not to have significant social relationships in the areas to which they have been relocated. Therefore, in temporary communities, "community organizing" was implemented [16]. Community organization is an intervention aimed at developing social connections and relationships among residents [17].

Regarding the form of support that was offered, Nitanai et al. [18], Nitanai and Goto [19], and Nakajima and Shiota [20] reported that disaster support teams from universities and NGOs implemented lunch or dinner gatherings in temporary communities to build a sense of connection among residents and to encourage the formation of community self-governing organizations. Furthermore, support teams from universities and NGOs assisted in holding and facilitating community meetings that were open for all community residents to participate in freely. These meetings, hosted by resident leaders to discuss community issues and improve the living environment, supported the establishment of a system where residents could solve problems themselves [18,19]. Nitanai et al. [18] reported that community-based activities by residents of temporary communities are enhanced by the abovementioned support for community organizing. This support might then be associated with more frequent implementation of community-based group exercises.

In light of these findings, supporting community organization may contribute to reducing these health risks in severely affected communities. Farra and Smith [21] indicated that public health nurses and other community and public health professionals should collaborate with communities to identify creative ways to reinstitute social ties as early as possible during the recovery period. Our findings support this perspective. In addition, a possible explanation for the lower frequency of group exercises in original communities is that these settings might have received less intensive support for the community compared with temporary communities. While our study did not directly measure support levels, these findings highlight the importance of ensuring that original communities are not overlooked in post-disaster support efforts. Therefore, community and public health professionals should actively promote community organization after disasters to reduce the risk of physical and mental health problems, not only in temporary communities but also in original communities.

This study has certain limitations. First, it entailed a cross-sectional design and took place two years and 10 months after the earthquake. Thus, it is impossible to determine the mechanism for the change in frequency of community-based activities entailed. For instance, our data were not capable of assessing the situation immediately after the relocation to temporary communities around September 2011. Second, community leaders were chosen as participants based on the assumption that they were likely to best understand their communities. Just how well they understood their population could not, however, be objectively determined. In the future, research that includes direct observation of the implementation of community activities after disasters is needed. Third, the response rate was 37.6% (32/85), introducing potential selection bias where leaders from more active communities might have been more likely to participate, possibly leading to an overestimation of exercise frequency. However, since this potential bias likely applied to both community types, the comparative findings between original and temporary settings are considered to maintain their significance.

Fourth, community-level demographics (e.g., age and gender) were not collected, which may have influenced exercise frequency. Collecting this information was particularly challenging due to the specific conditions in the disaster-affected areas of Otsuchi town. There was high population fluidity, where many residents remained registered in the community but actually lived with relatives outside the community, making it difficult even for leaders to accurately grasp the demographic profile. Additionally, ethical constraints prevented the linkage of survey data with public administrative records. Consequently, the lack of these variables and the small sample size precluded multivariate adjustments. Future research on a larger scale is necessary to confirm these findings and should explore ways to incorporate demographic factors while balancing respondent privacy and the unique field challenges of post-disaster settings. Furthermore, since multiple comparisons were performed without adjustment, some significant results might have been affected by inflated significance. These findings should be interpreted as preliminary trends rather than definitive causal explanations.

Fifth, we suggest the importance of support for community organization among communities affected by disasters. However, we did not investigate the relationship between the frequency of community-based group exercises and support for community organization. Therefore, a study focused on this relationship is also needed. Sixth, we did not investigate the relationship between the frequency of community activities and the health status of residents. Finally, a subset of participants (28.1%) provided information for both types of communities. While we treated these as independent observations based on the community-level analysis, we cannot entirely rule out the possibility that a single leader's reporting bias influenced the data for both community types. Despite these limitations, it is important to note that our findings revealed that community residents at newly established temporary communities implemented community-based activities more frequently than did those in original communities.

## Conclusions

These exploratory findings suggest that, approximately three years after the Great East Japan Earthquake, community-based group exercises were conducted significantly more frequently in temporary communities than in original communities within severely affected municipalities. This trend was particularly prominent in activities organized by the residents themselves. These findings suggest that residents in temporary settings may have demonstrated greater initiative in organizing community-based activities. This may be attributed to the severity of the disaster's impact and the support for community organizing provided during the recovery phase.

Our results underscore the vital importance of community organization as a public health intervention to mitigate the long-term risk of physical and mental health deterioration among disaster survivors. Public health nurses and healthcare practitioners should actively facilitate social ties and resident-led health activities. Crucially, such organizational support should be provided not only to visible temporary housing sites but also to original communities that may be at risk of being overlooked and receiving insufficient assistance.

## Appendices

Survey on Community Activities in Your Area

Instructions

Please select the most appropriate answer by marking a check (✓) or by writing your response in the space provided ( ).

Participation in this survey is voluntary. You may skip any questions you prefer not to answer; however, we would appreciate it if you could respond to as many questions as possible. Your participation and responses will not result in any disadvantage.

I. Temporary Housing Complex

Q1.

Are you responsible for a temporary housing complex as a community representative (e.g., neighborhood association president) or as a community welfare commissioner?

1. Yes

2. No

If you selected "Yes", go to Question 2.

If you selected "No", go to Question 14.

Q2.

In which district is the temporary housing complex you are responsible for located?

1. District A

2. District B

3. District C

4. District D

Q3.

How long have you been serving as a representative in your current area? (Approximate answers are acceptable.)

(   ) years and (   ) months

Q4.

During January 2014, how frequently were community-based group activities held in the temporary housing complex you are responsible for?

Note: Community-based group activities refer to events held in local parks, squares, community centers, or meeting halls in which multiple residents participate together. Examples include physical activities (e.g., morning radio calisthenics), social gatherings (e.g., tea parties or shared meals), hobby activities (e.g., crafts, computers, or karaoke), neighborhood or residents' association activities and study sessions, and local festivals.

1. Almost every day

2. About 3-4 times a week

3. About 1-2 times a week

4. About 2-3 times a month

5. About once a month

6. Not at all

Q5.

Compared with January 2013, did the number of community-based group activities in January 2014 increase or decrease in the temporary housing complex you are responsible for?

1. Increased -> Go to Question 6

2. Decreased -> Go to Question 7

3. No change -> Go to Question 8

Q6.

Please answer this question only if you selected "1. Increased" in Question 5.

Compared with January 2013, why did the number of community-based group activities increase in January 2014 in the temporary housing complex you are responsible for? (Select all that apply.)

1. Efforts by neighborhood or residents' associations

2. Efforts by community welfare commissioners

3. Presence of key individuals within the housing complex who lead activities

4. Efforts by residents

5. Support from community support workers and life support advisors (LSAs)

6. Support from external organizations (e.g., NGOs, universities)

7. Support from the municipal government

8. Other (     )

-> Go to Question 8.

Q7.

Please answer this question only if you selected "2. Decreased" in Question 5.

Compared with January 2013, why did the number of community-based group activities decrease in January 2014 in the temporary housing complex you are responsible for? (Select all that apply.)

1. Decrease in the number of residents (e.g., due to relocation)

2. Decrease in the number of participants

3. Absence of key individuals who previously led activities (e.g., due to illness or injury)

4. Lack of a suitable venue for activities

5. Reduced support from community support workers and life support advisors (LSAs)

6. Reduced support from external organizations (e.g., NGOs, universities)

7. Reduced support from the municipal government

8. Other (     )

II. Community-Based Group Exercises in Temporary Housing Complexes

Note: Community-based group exercises (e.g., calisthenics) include not only those centered on physical movement, such as morning radio calisthenics, exercise classes, or group walking, but also primarily social activities (e.g., tea gatherings) if they include physical exercise (e.g., health calisthenics) at the end. Please count all such activities as "activities involving physical exercise".

Q8.

Compared with January 2013, did the number of community-based group exercises in January 2014 increase or decrease in the temporary housing complex you are responsible for?

1. Increased

2. Decreased

3. No change

Q9.

During January 2014, how often were community-based group exercises held in the temporary housing complex you are responsible for?

1. Almost every day

2. About 3-4 times a week

3. About 1-2 times a week

4. About 2-3 times a month

5. About once a month

6. Not at all

If you selected "6. Not done", go to Question 14.

If you selected "1-5", go to Question 10.

Q10.

During January 2014, how often were community-based group exercises in the temporary housing complex you are responsible for organized by each of the following groups? Please answer for each.

Note: If two or more organizations or groups jointly conducted a single activity, please count it as one instance for each group.

Example: If a neighborhood association and an external organization (e.g., an NGO or a university) jointly held an exercise class, please count it as one instance under both "Activities organized by neighborhood associations" and "Activities organized by external organizations (e.g., NGOs or universities)".

(1) Activities organized by neighborhood associations

1. Almost every day

2. About 3-4 times a week

3. About 1-2 times a week

4. About 2-3 times a month

5. About once a month

6. Not at all

(2) Activities organized by resident groups that are not neighborhood or residents' associations

1. Almost every day

2. About 3-4 times a week

3. About 1-2 times a week

4. About 2-3 times a month

5. About once a month

6. Not at all

(3) Activities organized by community support staffs (CSSs), life support advisors (LSAs), and related personnel

1. Almost every day

2. About 3-4 times a week

3. About 1-2 times a week

4. About 2-3 times a month

5. About once a month

6. Not at all

(4) Activities organized by the municipal government or organizations commissioned by it

1. Almost every day

2. About 3-4 times a week

3. About 1-2 times a week

4. About 2-3 times a month

5. About once a month

6. Not at all

(5) Activities organized by external organizations (e.g., NGOs, universities)

1. Almost every day

2. About 3-4 times a week

3. About 1-2 times a week

4. About 2-3 times a month

5. About once a month

6. Not at all

(6-1) Activities organized by other organizations, groups, or individuals

1. Almost every day

2. About 3-4 times a week

3. About 1-2 times a week

4. About 2-3 times a month

5. About once a month

6. Not at all

(6-2) What type of organization, group, or individual organized these activities? (     )

Q11.

On average, how many people participated in community-based group exercises in the temporary housing complex you are responsible for during January 2014?

1. 1-3 participants

2. 4-6 participants

3. 7-9 participants

4. 10-12 participants

5. 13-15 participants

6. 16 or more participants

Q12.

During January 2014, what types of exercises were included in community-based group exercises in the temporary housing complex you are responsible for? (Select all that apply.)

1. Instructor-led health exercises

2. Radio calisthenics performed without an instructor

3. Other health exercises performed without an instructor (excluding radio calisthenics)

4. Instructor-led exercise classes (e.g., walking, Nordic walking)

5. Resident-led exercise activities (e.g., walking, Nordic walking)

6. Instructor-led sports classes (e.g., golf, baseball)

7. Resident-led sports activities (e.g., golf, baseball)

8. Other (     )

Q13.

During January 2014, what difficulties did you encounter regarding community-based group exercises in the temporary housing complex you are responsible for? (Select all that apply.)

1. Low participation in activities

2. Participation is limited to the same individuals

3. Lack of coordinators to organize activities (e.g., scheduling time and location)

4. Lack of a suitable venue for activities

5. Lack of necessary supplies or funding

6. Lack of instructors for physical activities (e.g., calisthenics or sports)

7. Uncertainty about what types of activities to organize

8. No difficulties

9. Other (     )

III. Original Communities (Less Affected by the Earthquake)

Q14.

Are you responsible for an original community as a community representative (e.g., neighborhood association president) or as a community welfare commissioner?

1. Yes

2. No

If you selected "2. No", this concludes the survey.

Thank you very much for your cooperation.

If you selected "1. Yes" in Question 14, please answer Questions 15 and beyond.

Q15.

In which district is the original community you are responsible for located?

1. District A

2. District B

3. District C

4. District D

Q16.

How long have you been serving as a representative in your current area? (Approximate answers are acceptable.)

(   ) years and (   ) months

Q17.

During January 2014, how frequently were community-based group activities held in the original community you are responsible for?

Note: Community-based group activities refer to events held in local parks, squares, community centers, or meeting halls in which multiple residents participate together. Examples include physical activities (e.g., morning radio calisthenics), social gatherings (e.g., tea parties or shared meals), hobby activities (e.g., crafts, computers, or karaoke), neighborhood or residents' association activities and study sessions, and local festivals.

1. Almost every day

2. About 3-4 times a week

3. About 1-2 times a week

4. About 2-3 times a month

5. About once a month

6. Not at all

Q18.

Compared with January 2013, did the number of community-based group activities in January 2014 increase or decrease in the original community you are responsible for?

1. Increased -> Go to Question 19

2. Decreased -> Go to Question 20

3. No change -> Go to Question 21

Q19.

Please answer this question only if you selected "1. Increased" in Question 18.

Compared with January 2013, why did the number of community-based group activities increase in January 2014 in the original community you are responsible for? (Select all that apply.)

1. Efforts by neighborhood or residents' associations

2. Efforts by community welfare commissioners

3. Presence of key individuals within the community who lead activities

4. Efforts by residents

5. Support from community support workers and life support advisors (LSAs)

6. Support from external organizations (e.g., NGOs, universities)

7. Support from the municipal government

8. Other (     )

-> Go to Question 21.

Q20.

Please answer this question only if you selected "2. Decreased" in Question 18.

Compared with January 2013, why did the number of community-based group activities decrease in January 2014 in the original community you are responsible for? (Select all that apply.)

1. Decrease in the number of residents (e.g., due to relocation)

2. Decrease in the number of participants

3. Absence of key individuals who previously led activities (e.g., due to illness or injury)

4. Lack of a suitable venue for activities

5. Reduced support from community support workers and life support advisors (LSAs)

6. Reduced support from external organizations (e.g., NGOs, universities)

7. Reduced support from the municipal government

8. Other (     )

-> Go to Question 21.

Q21.

Compared with the period before the earthquake, did the number of community-based group activities in January 2014 increase or decrease in the community you are responsible for?

1. Increased -> Go to Question 22.

2. Decreased -> Go to Question 23.

3. No change -> Go to Question 24.

Q22.

Please answer this question only if you selected "1. Increased" in Question 21.

Compared with the period before the earthquake, why did the number of community-based group activities increase in January 2014 in the original community you are responsible for? (Select all that apply.)

1. Efforts by neighborhood or residents' associations

2. Efforts by community welfare commissioners

3. Presence of key individuals within the community who lead activities

4. Efforts by residents

5. Support from community support workers and life support advisors (LSAs)

6. Support from external organizations (e.g., NGOs, universities)

7. Support from the municipal government

8. Other (     )

-> Go to Question 24.

Q23.

Please answer only if you selected "2. Decreased" in Question 21.

Compared with the period before the earthquake, why did the number of community-based group activities decrease in January 2014 in the original community you are responsible for? (Select all that apply.)

1. Decrease in the number of residents (e.g., due to relocation)

2. Decrease in the number of participants

3. Absence of key individuals who previously led activities (e.g., due to illness or injury)

4. Lack of a suitable venue for activities

5. Lack of necessary supplies or funding

6. Self-restraint of activities due to the disaster

7. Reduced support from the municipal government

8. Other (     )

-> Go to Question 24.

IV. Community-Based Group Exercises in Original Communities

Q24.

Compared with January 2013, did the number of community-based group exercises in January 2014 increase or decrease in the original community you are responsible for?

1. Increased

2. Decreased

3. No change

Q25.

During January 2014, how often were community-based group exercises held in the original community you are responsible for?

1. Almost every day

2. About 3-4 times a week

3. About 1-2 times a week

4. About 2-3 times a month

5. About once a month

6. Not at all

This concludes the questions for those who selected "6. Not performed".

Thank you very much for your cooperation despite your busy schedule.

If you selected "1-5", please go to Question 26.

Q26.

During January 2014, how often were community-based group exercises in the original community you are responsible for organized by each of the following groups? Please answer for each.

Note: If two or more organizations or groups jointly conducted a single activity, please count it as one instance for each group.

Example: If a neighborhood association and an external organization (e.g., an NGO or a university) jointly held an exercise class, please count it as one instance under both "Activities organized by neighborhood associations" and "Activities organized by external organizations (e.g., NGOs or universities)".

(1) Activities organized by neighborhood associations

1. Almost every day

2. About 3-4 times a week

3. About 1-2 times a week

4. About 2-3 times a month

5. About once a month

6. Not at all

(2) Activities organized by resident groups (e.g., clubs) that are not neighborhood or residents' associations

1. Almost every day

2. About 3-4 times a week

3. About 1-2 times a week

4. About 2-3 times a month

5. About once a month

6. Not at all

(3) Activities organized by community support staffs (CSSs), life support advisors (LSAs), and related personnel

1. Almost every day

2. About 3-4 times a week

3. About 1-2 times a week

4. About 2-3 times a month

5. About once a month

6. Not at all

(4) Activities organized by the municipal government or organizations commissioned by it

1. Almost every day

2. About 3-4 times a week

3. About 1-2 times a week

4. About 2-3 times a month

5. About once a month

6. Not at all

(5) Activities organized by external organizations (e.g., NGOs, universities)

1. Almost every day

2. About 3-4 times a week

3. About 1-2 times a week

4. About 2-3 times a month

5. About once a month

6. Not at all

(6-1) Activities organized by other organizations, groups, or individuals

1. Almost every day

2. About 3-4 times a week

3. About 1-2 times a week

4. About 2-3 times a month

5. About once a month

6. Not at all

(6-2) What type of organization, group, or individual organized these activities? (     )

Q27.

On average, how many people participated in community-based group exercises in the original community you are responsible for during January 2014?

1. 1-3 participants

2. 4-6 participants

3. 7-9 participants

4. 10-12 participants

5. 13-15 participants

6. 16 or more participants

Q28.

During January 2014, what types of exercises were included in community-based group exercises in the original community you are responsible for? (Select all that apply.)

1. Instructor-led health exercises

2. Radio calisthenics performed without an instructor

3. Other health exercises performed without an instructor (excluding radio calisthenics)

4. Instructor-led exercise classes (e.g., walking, Nordic walking)

5. Resident-led exercise activities (e.g., walking, Nordic walking)

6. Instructor-led sports classes (e.g., golf, baseball)

7. Resident-led sports activities (e.g., golf, baseball)

8. Other (     )

Q29. During January 2014, what difficulties did you encounter regarding community-based group exercises in the original community you are responsible for? (Select all that apply.)

1. Low participation in activities

2. Participation is limited to the same individuals

3. Lack of coordinators to organize activities (e.g., scheduling time and location)

4. Lack of a suitable venue for activities

5. Lack of necessary supplies or funding

6. Lack of instructors for physical activities (e.g., calisthenics or sports)

7. Uncertainty about what types of activities to organize

8. No difficulties

9. Other (     )

This concludes the survey.

Thank you very much for taking the time to participate.
